# An innovative technique for closing a post-endoscopic submucosal dissection ulcer to prevent early dehiscence, the muscle-grasping clip with line pulley securing technique

**DOI:** 10.1055/a-2550-4091

**Published:** 2025-03-21

**Authors:** Shinya Nagae, Ryoju Negishi, Kohei Ono, Yohei Minato, Deepak Madhu, Hideyuki Chiba, Ken Ohata

**Affiliations:** 1 215674 Gastroenterology, Itabashi Chuo Medical Center, Tokyo, Japan; 2 13635 Gastrointestinal Endoscopy, NTT Medical Center Tokyo, Tokyo, Japan; 3 29290 Gastroenterology, Lisie Hospital, Kochi, India; 4 74155 Gastroenterology, Omori Red Cross Hospital, Tokyo, Japan

Endoscopic submucosal dissection (ESD) is a standard treatment for early gastrointestinal cancer, but carries risks of delayed bleeding and perforation. Endoscopic closure of the post-ESD ulcer can reduce these risks 1 . We previously reported the “clip with line pulley securing” (CLiPS) technique 2 3 4 , a simple cost-effective method that allows closure without scope reinsertion. Mucosa-to-mucosa closure may however result in early dehiscence, particularly in large mucosal defects. To address this limitation, we developed a modified technique, the muscle-grasping CLiPS (M-CLiPS) technique ( Fig. 1 ).

**Fig. 1:**
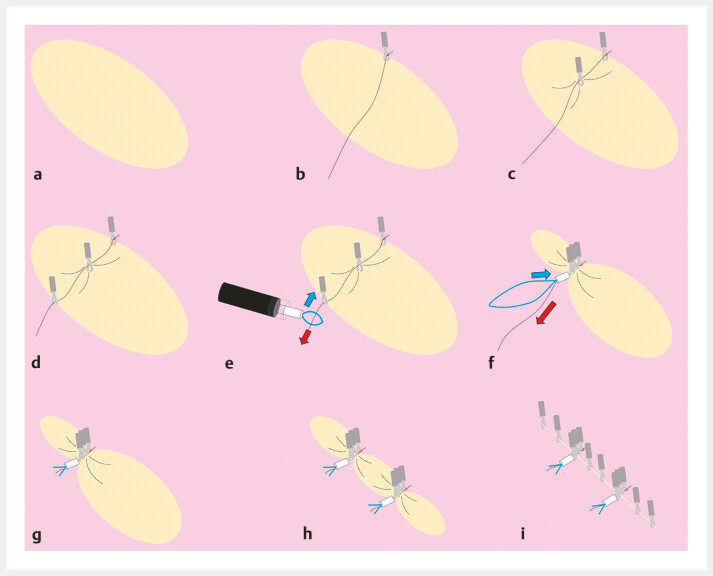
Schematic of the muscle-grasping clip with line pulley securing (M-CLiPS) technique showing: **a** a post-endoscopic submucosal dissection ulcer defect; **b** a reopenable clip with an attached string placed at the edge of the distal end of the defect; **c** another clip attached across the string in the muscle layer at the center of the ulcer; **d** a third clip placed at the edge of the proximal end; **e** a plastic detachable snare inserted through the scope channel and positioned over the line; **f** tightening of the snare to form a knot; **g** cutting of the excess strings; **h** a second M-CLiPS procedure at the other side of the lesion; **i** additional through-the-scope hemostatic clips deployed to complete the closure.

A 64-year-old man underwent ESD for a 40-mm early gastric cancer at the lesser curvature of the gastric body, resulting in a 50-mm ulcer. As the patient was on antithrombotic therapy, mucosal defect closure was performed to prevent bleeding, with the M-CLiPS technique used. A clip (Sure Clip; Micro-Tech Co., Ltd, Nanjing, China) with an attached string (Bear nylon monofilament suture; BEAR Medic Co, Ibaraki, Japan) was placed on the distal mucosal edge of the ulcer. Another clip was deployed to grasp the muscle layer at the center, followed by clipping of the proximal mucosal edge. Subsequently, a plastic detachable snare (Polyloop; Olympus, Tokyo, Japan) was inserted over the string through the instrument channel. The string was pulled to approximate all of the clips, achieving closure of the mucosa and muscle. Because of the large size of the ulcer, the M-CLiPS technique was performed at a second site to reduce the ulcer size. Additional clips were then deployed to secure the edges ( Fig. 2 ; Video 1 ). The procedure lasted 40 minutes. Follow-up endoscopy on postoperative day 2 confirmed intact apposition with no dehiscence ( Fig. 3 ). The patient was discharged on postoperative day 3 without experiencing any complications.

**Fig. 2:**
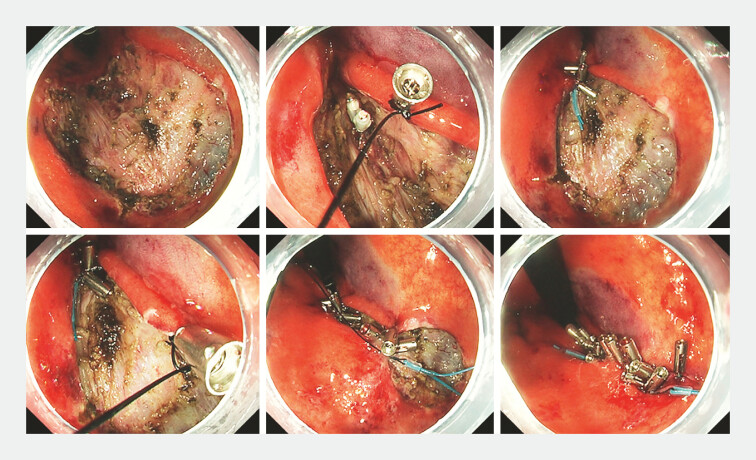
Endoscopic images of the muscle-grasping clip with line pulley securing (M-CLiPS) technique used for a post-endoscopic submucosal dissection ulcer.

Demonstration of the muscle-grasping clip with line pulley securing (M-CLiPS) technique in a patient on antithrombotic therapy with a 50-mm post-endoscopic submucosal dissection ulcer.Video 1

**Fig. 3:**
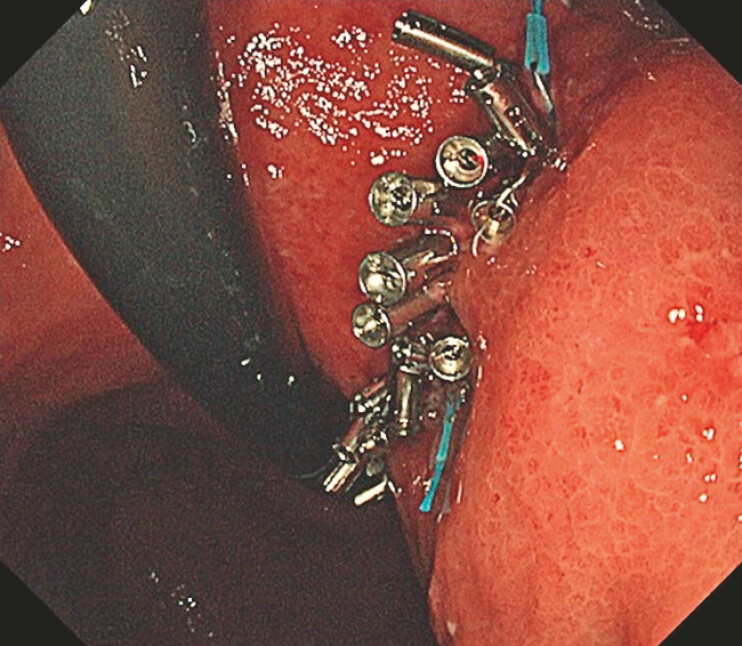
Endoscopic appearance 2 days after the muscle-grasping clip with line pulley securing (M-CLiPS) procedure had been performed.

The M-CLiPS technique achieves robust double-layered closure by incorporating the muscle layer and eliminating submucosal dead space ( Fig. 4 ). This ensures stable closure, reduces the risk of early dehiscence, and prevents delayed bleeding and perforation. This technique offers a more effective approach for large post-ESD ulcers compared with the conventional CLiPS technique.

**Fig. 4:**
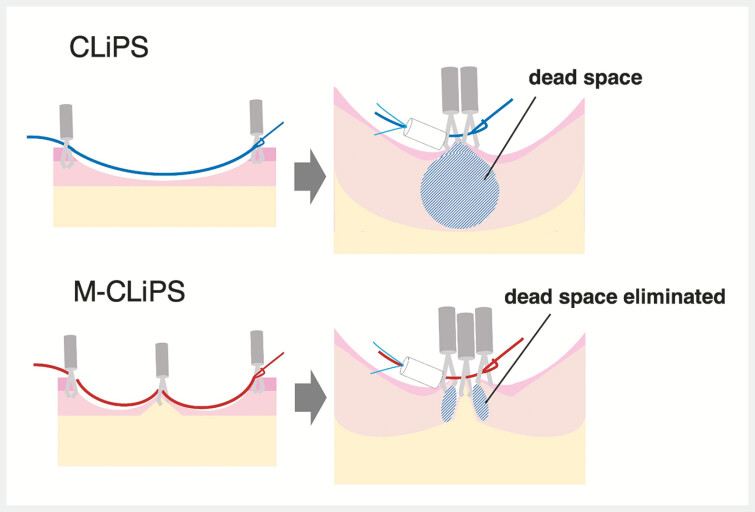
Schematic illustrating the difference between the clip with line pulley securing (CLiPS) and muscle-grasping CLiPS (M-CLiPS) techniques.

Endoscopy_UCTN_Code_TTT_1AO_2AZ

## Notes

Endoscopy E-Videos https://eref.thieme.de/e-videos
*E-Videos* is an open access online section of the journal *Endoscopy* , reporting on interesting cases and new techniques in gastroenterological endoscopy. All papers include a high-quality video and are published with a Creative Commons CC-BY license. Endoscopy E-Videos qualify for HINARI discounts and waivers and eligibility is automatically checked during the submission process. We grant 100% waivers to articles whose corresponding authors are based in Group A countries and 50% waivers to those who are based in Group B countries as classified by Research4Life (see: https://www.research4life.org/access/eligibility/ ). This section has its own submission website at https://mc.manuscriptcentral.com/e-videos .
